# Dung beetle (Coleoptera: Scarabaeoidea) assemblages in the western Italian Alps: benchmark data for land use monitoring

**DOI:** 10.3897/BDJ.4.e10059

**Published:** 2016-11-28

**Authors:** Claudia Tocco, Martin H Villet

**Affiliations:** ‡ Department of Zoology and Entomology, Rhodes University, Grahamstown, South Africa; § Department of Entomology and Arachnology, Albany Museum,, Grahamstown, South Africa

**Keywords:** Dung beetles, pitfall traps, Scarabaeinae, Aphodiinae, Geotrupidae, Italian Alps, seasonality.

## Abstract

**Background:**

Traditional agro-pastoral practices are in decline over much of the Alps (MacDonald et al. 2000), leading to shrub and tree encroachment, and this represents one of the main threats for the conservation of alpine biodiversity, as many plant and animal species are dependent on the presence of semi-natural open habitats. However, quantifying this environmental change and assessing its impact on biodiversity may be difficult, especially in the context of sparse historical survey data. The accessibility of contemporary data about local biodiversity surveys in general, and indicator taxa in particular, is an essential consideration for planning future evaluations of conservation status in the Alps and for conservation plans that use ecological indicators to monitor temporal changes in biodiversity. Dung beetles are important ecosystem service providers (Nichols et al. 2008) that have been assessed as a good ecological indicator taxon in several studies (reviewed by Nichols and Gardner 2011), and although the Alps is perhaps one of the best-studied regions in respect of dung beetles, there are still only eight readily-accessible publications. We have augmented and comprehensively reviewed the data from these publications.

**New information:**

We first provide data about changes on a temporal scale of seasons in a dung beetle community in the western Italian Alps, an issue that has to be addressed in the local assemblages because it would affect regional biomonitoring and conservation research. This survey of 12 099 individuals belonging to 22 species illustrates a distinct seasonal pattern at a single site. Second, we collate the results of 13 published surveys of the presence of 46 species of dung beetles in 11 valleys in the western Italian Alps in the period from 2005 to 2012, a period of accelerated change in land use that started around 1945 (MacDonald et al. 2000). Because ten of the surveys used baited pitfall traps and four more used manual collection of specimens, the abundance data were not strictly comparable and they were therefore transformed to binary data (presence-or-absence records) with measures of sampling effort. The results illustrate both spatial variation and temporal variation at the scale of years.Because of the importance of dung beetles in agro-pastoral ecosystems and the high sensitivity of montane ecosystems to climate change, these spatially and temporally explicit data sets provide important baseline information about western Italian Alpine dung beetles for investigations of the effects of land use change under ongoing climate change scenarios.

## Introduction

Traditional agro-pastoralism is declining over much of the Alps since 1945 (MacDonald et al. 2000), leading to the encroachment of woody vegetation that is a primary threat for conservation of alpine biodiversity because many species depend on the presence of semi-natural open habitats (Chamberlain et al. 2013, Marini et al. 2009, Maurer et al. 2006). Appropriate means of monitoring changes in biodiversity are important in these environments as a part of managing the ecological services provided by alpine communities.

Many of the environmental effects produced by livestock are mediated by dung beetles' activities. Dung beetles are coprophagous members of the Scarabaeinae, Aphodiinae and Geotrupidae and are the dominant faunal component of vertebrate dung. Dung beetles bring many benefits to animal health and human welfare, especially in agro-environmental contexts, by manipulating livestock faeces during their feeding and nesting activities, and thus providing ecosystem functions including dung removal; relocation of nutrients into the soil; enhancement of soil porosity, aeration and water infiltration; and control of the abundance of dung-breeding flies and dung-dispersed nematodes and protozoa (Nichols et al. 2008, Spector 2006). Dung beetles also satisfy biological and logistical criteria for the selection of bioindicators (e.g McGeoch 1998) and they are assessed as a good ecological indicator group (Nichols and Gardner 2011).

Several studies have demonstrated the key role of tropical dung beetle assemblages as ecological indicators, whilst numerous others have described local dung beetle communities at temperate latitudes in Europe with particular focus on montane areas in France, but there is a paucity of studies on dung beetle communities in the Italian Alps. We synthesize data from published studies carried out in the western Italian Alps in the last decade (Chamberlain et al. 2015, Tocco et al. 2013a, Tocco et al. 2013b, Negro et al. 2011a, Macagno and Palestrini 2009, Palestrini et al. 2008a, Palestrini et al. 2008b) with new survey data from the same region. The new survey data that show that season may be an important variable in future surveys of dung beetle communities’ conservation status and their use as ecological indicators (cf. Tocco et al. 2013a). They also suggest that surveys should rely less on quantitative data about abundance, which may change with season and weather, and more on binary data about presence or absence, which are more robust to such fluctuations. For this reason, we have tabulated the published data as binary presence / absence data (Table 3).

## Sampling methods

### Study extent

To assess the effects of seasonality on alpine dung beetle assemblages, data were collected from a site in the Troncea valley (termed the Troncea B site) in the western Italian Alps over four occasions in 2011.

**Figure 1.**
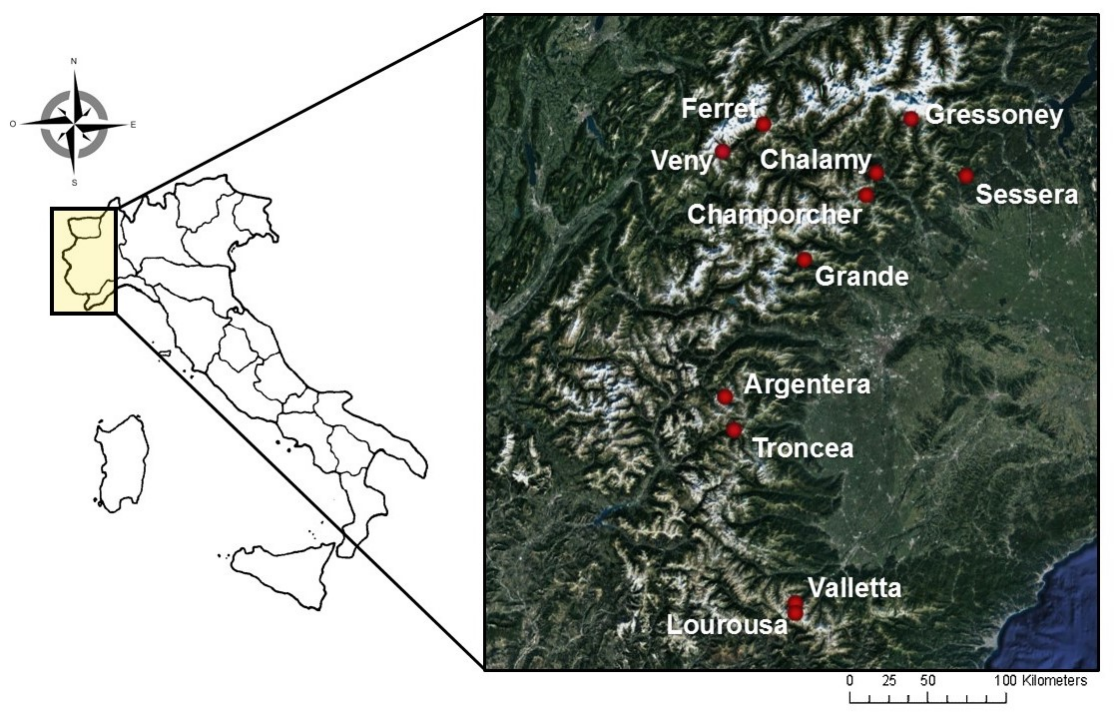
Illustration of the eleven valleys in the western Italian Alps where dung beetle surveys were carried out.

Historically, surveys specifically focused on dung beetles in the western Italian Alps have occurred a total of thirteen occasions, spread unevenly across eleven valleys (the Argentera, Chalamy, Champorcher, Ferret, Gressoney, Grande, Lourousa, Sessera, Troncea, Valletta and Veny valleys) and the years 2005 to 2012 (Chamberlain et al. 2015, Tocco et al. 2013a, Tocco et al. 2013b, Negro et al. 2011a, Negro et al. 2011b, Macagno and Palestrini 2009, Palestrini et al. 2008a, Palestrini et al. 2008b) (Fig. 1). The location and climatic characteristics of the valleys are summarised in (Table 1).

### Sampling description

Seasonal sampling at Troncea B was carried out using pitfall traps with bait suspended over them using a tripod (Tocco et al. 2013a, Tocco et al. 2013b,Tocco et al. 2016). Each trap consisted of a 1.5 l clear plastic bottle, 9 cm in diameter, cut horizontally about 25 cm from its bottom: the top was cut and inverted to make a funnel that was inserted into the main 25 cm container. The bait, 200 g of fresh cow dung, was wrapped in gauze and suspended over the trap just above the funnel using a tripod made with three 50 cm-long sticks. A standard mixture of water, liquid soap (to reduce surface tension) and sodium chloride was used as a preserving fluid (Tocco et al. 2013a, Tocco et al. 2013b, Tocco et al. 2016). Trapped beetles were preserved, taken to the laboratory, dried, counted and identified to species level using dichotomous keys (Dellacasa and Dellacasa 2006, Paulian and Baraud 1982). Examination of aedeagus was necessary in the case of groups containing cryptic species (Dellacasa and Dellacasa 2006).

Four early published surveys carried out in the Chalamy, Champorcher, Ferret and Veny valleys in 2005-2006 used active manual collecting of dung beetles from dung pads (Table 2). Ten later surveys in the Argentera, Chalamy, Ferret, Gressoney, Grande, Lourousa, Sessera, Troncea, Valletta and Veny valleys used pitfall traps baited with cow dung. The sampling effort (numbers of sampling sites, traps and events) reported in the original publications of each survey is collated in (Table 2). The surveys were all carried out in summer (June to September), corresponding to the activity period of adult Alpine dung beetles, on 1-19 occasions within a year, over one or two years, depending on the particular survey. Because the surveys used different survey methods, their abundance data were not strictly comparable and their results were therefore transformed to binary data (presence-or-absence records) with measures of sampling effort and collated into a single dataset.

## Geographic coverage

### Description

**Argentera valley (Piemonte, Italy)**: larch forest (*Larix decidua* Miller), shrub (*Rhododendron ferrugineum* L), alpine meadow (graminaceous plant) and grassland (graminaceous plant). Elevations ranged from 2203 to 2500 m a.s.l. **Chalamy valley (Valle d’Aosta, Italy)**: beech forest (*Fagus sylvatica* L.), Scots pine forest (*Pinus sylvestris* L.), mountain pine forest (*Pinus uncinata* Mill.) and pasture (graminaceous plant). Elevations ranged from 1002 to 1997 m a.s.l. **Champorcher valley (Valle d’Aosta, Italy)**: pasture (graminaceous plant). Elevation ranged from 1645 to 2584 m a.s.l. **Ferret valley (Valle d’Aosta, Italy)**: Coniferous forests (dominated by larch, *Larix decidua*), wet meadows, shrub (*Rhododendron ferrugineum* L. and *Vaccinium myrtillus* L.), and anthropogenic woods for picnickers and pasture. Elevation ranged from 1500 to 2062 m a.s.l. **Grande valley (Piemonte, Italy)**: forest, shrub, alpine meadow, and grassland. Elevation ranged from 1753 to 2180 m a.s.l. **Gressoney valley (Valle d’Aosta, Italy)**: larch forest, shrub (*Juniperus nana* Willd), alpine meadow, and grassland. Elevation ranged from 1959 to 2772 m a.s.l. **Lourousa and Valletta valleys (Piemonte, Italy)**: pasture dominated by Festuca gr. ovina, *Festuca scabriculmis*, and *Rumex alpinus*. Elevation of Lourosa and Valletta sampling site was 1959 and 1743 m a.s.l., respectively. **Sessera valley (Piemonte, Italy)**: beech forest, pioneer forest (*Picea abies* L.), shrub (*Rhododendron ferrugineum* L) and pasture. Elevation ranged from 1000 to 1600 m a.s.l. **Troncea valley (Piemonte, Italy)**: shrub (*Rhododendron ferrugineum* L and *Juniperus nana* Willd). Elevations ranged from 1960 to 2360 m a.s.l. **Veny valley (Valle d’Aosta, Italy)**: pasture. Elevation ranged from 1550 to 2200 m a.s.l.

**Coordinates**

**Argentera valley**: 44°54'N; 6°54'E. **Chalamy valley**: 45°41'N; 7°38'E. **Champorcher valley**: 45°36'N; 7°34'E. **Ferret valley**: 45°50'N; 7°01'E. **Gressoney valley**: 45°51'N; 7°48'E. **Grande valley**: 45°22'N; 7°16'E. **Lourousa valley**: 44°12'N; 7°16'E. **Sessera valley**: 45°40'N; 8°05´E. **Troncea valley**: 44° 57’N, 6°. 57’E. **Valletta valley**: 44°10'N; 7°16'E. **Veny valley**: 45°46'N; 6°52'E.

### Coordinates

 and Latitude; and Longitude.

## Taxonomic coverage

### Description

The published surveys collectively report 46 species of dung beetle inhabiting eleven valleys of the western Italian Alps, surveyed irregularly over seven years Table 3. The communities in all valleys were dominated by Aphodiinae Leach, 1815, both by abundance and by species richness. Geotrupidae Latreille, 1802 and Scarabaeidae Latreille, 1802 form a smaller part of the communities, the proportion depending on the valley. Our nomenclature follows Dellacasa and Dellacasa (2016). In the table we listed *Onthophagus vacca* Linnaeus recorded by Macagno and Palestrini (2009) from Chalamy valley, but this record my pertain to *Onthophagus medius* (Kugelann) (see Rössner et al. 2010).

The Troncea B survey collected a total of 22 dung beetle species belonging to the Scarabaeidae (Aphodiinae and Scarabaeinae) and Geotrupidae (Suppl. material 1). Voucher material is deposited in the Department of Life Sciences and System Biology, University of Torino, Italy.

## Temporal coverage

**Data range:** 2005 6 01 – 2012 9 30.

### Notes

**Argentera, Grande and Gressoney valleys**: trapping was carried out during four sampling occasions in August and September 2010. **Chalamy valley**: trapping lasted from July to September 2007, and all traps were emptied and re-baited every week for a total of eleven sampling occasions. **Champorcher valley**: active manual collection was carried out in an unspecified number of sampling occasions in 2005 and 2006. **Ferret valley**: active manual collection occurred on 23 sampling occasions from June to September 2005, and trapping was carried out from June to October 2007 for a total of nineteen sampling occasions. **Lourousa and Valletta valleys**: trapping ran from June to September 2008, and traps were emptied every 3wk for a total of 5 sampling occasions. **Sessera valley**: trapping lasted from early June to late September 2010, all traps were emptied and re-baited every 3 weeks for a total of 5 sampling occasions. **Troncea valley**: trapping occurred on four occasion from July to September 2011 (Troncea B), and on six occasion from June to September 2011 and 2012. **Veny valley**: active manual collection occurred from June to September 2005 on 12 sampling occasions.

## Usage rights

### Use license

Creative Commons Public Domain Waiver (CC-Zero)

### IP rights notes

These data can be freely used, provided their source is cited.

## Data resources

### Data package title

Dung beetles of Troncea B

### Number of data sets

1

### Data set 1.

#### Data set name

Troncea B

#### Number of columns

33

#### Description

Dung beetles collected from a site in the Troncea valley (termed the Troncea B site) in the western Italian Alps over four occasions in 2011.

**Data set 1.** 

Column label	Column description
Date	Date of the sample collection
Valley	Name of the sampling area
Year	Year of sample collection
Month	Month of sample collection
Sampling occasion	Code of sampling occasion
Dominant plant species	Dominat plant species of the sample site
Altitude	Altitude in metre of the sample site
Site	Code of the sample site
Number of traps	Number of active traps
Abundance	Total dung beetle abundance
Species richness	Number of dung beetle species
Acrossus depressus (Kugelann)	Dung beetle species
Acrossus rufipes (Linnaeus)	Dung beetle species
Agoliinus satyrus (Reitter)	Dung beetle species
Amidorus immaturus (Mulsant)	Dung beetle species
Amidorus obscurus (Mulsant & Rey)	Dung beetle species
Anoplotrupes stercorosus (Scriba)	Dung beetle species
Aphodius fimetarius (Linnaeus)	Dung beetle species
Bodilopsis rufa (Moll)	Dung beetle species
Colobopterus erraticus (Linnaeus)	Dung beetle species
Coprimorphus scrutator (Herbst)	Dung beetle species
Esymus pusillus (Herbst)	Dung beetle species
Euheptaulacus carinatus (Germar)	Dung beetle species
Geotrupes stercorarius (Linnaeus)	Dung beetle species
Onthophagus baraudi (Nicolas)	Dung beetle species
Onthophagus fracticornis (Preyssler)	Dung beetle species
Oromus alpinus (Scopoli)	Dung beetle species
Otophorus haemorrhoidalis (Linnaeus)	Dung beetle species
Parammoecius corvinus (Erichson)	Dung beetle species
Planolinus fasciatus (Olivier)	Dung beetle species
Rhodaphodius foetens (Fabricius)	Dung beetle species
Teuchestes fossor (Linnaeus)	Dung beetle species
Trypocopris alpinus (Sturm & Hagenbach)	Dung beetle species

## Additional information

**Seasonality effect**

Dung beetles collected during the Troncea B survey were classified according to their nesting guilds (Halffter and Edmonds 1982) to calculate the endocoprids and paracoprid abundance. Principal component analysis (PCA) was used to evaluate the dung beetle assemblage variation among sampling occasions using total abundance, species richness, endocoprid and paracoprids abundance as variables because these are the variables that are usually measured to quantify biological diversisty during biomonitoring. In the analysis, trap was used as sampling unit. Analysis were performed using the *stats* package (version 3.2.3) and the results were visualized using the *ggfortify* package (version 0.2.0.) (Horikoshi and Tang 2016), both run in R 3.2.3 (R Development Core Team 2011).

**Figure 2.**
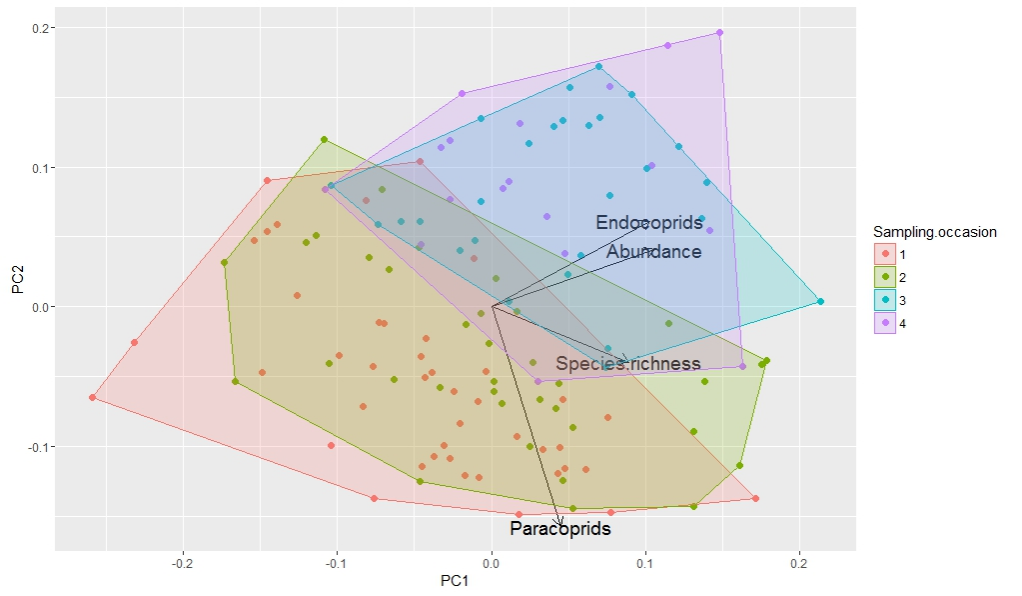
Scatterplots of the first two components scores obtained from the PCA of dung beetle diversity of Troncea B (percentages of the explained variance: PC1= 64%, PC2= 25%), of first sampling occasion (red), second sampling occasion (green), third sampling occasion (light blue), and fourth sampling occasion (purple).

The ordination showed seasonal variation in the biomonitoring variables describing the assemblage. A plot of the first two components (Fig. 2) and an examination of the associated coefficients of the eigenvectors show a bbuild-up of specimens and species as the season porgressed (along the first principle component) and a change in the dominant ecological guild from paracoprids (Onthophagini and Geotrupidae) early in the sampling period to endocoprids (Aphodiinae) later in the sampling period. The assemblages collected during the two early visits overlapped extensively, as did the assemblages from the late visits, but the two periods were somewhat distinct. This implies that samples collected for biomonitoring must cover the entire season of activity of the dung beetle community if they are to be comparable between sites and years.

The samples collected with pit traps from Troncea valley in different years contained the same set of species, with only one unique species in each year. The results from different years from the Chalamy and Ferret vallies cannot be compared rigorously because they were obtained with different collecting techniques (Table 2).

## Supplementary Material

Supplementary material 1 Dung beetles of Troncea B Data type: occurrences
Brief description: Sampling at Troncea valley (site B) carried out from June to September 2011.
File: oo_107088.xlsx Tocco C. and Villet M.H.

## Figures and Tables

**Table 1.** Climatic characteristics of the eleven valleys of the western Italian Alps surveyed from 2005 to 2012 (ARPA 2016).

**Valley**	**GPS**	**Mean annual air temperature (°C)**	**Mean air temperature (°C)**		**Mean annual precipitation (mm)**
			**Coldest month**	**Warmest month**	
**Argentera**	44°54'N; 6°54'E	4.9	January: -2.7	July: 13.4	1123
**Chalamy**	45°41'N; 7°38'E	9.3	January: -0.3	July: 18.9	877
**Champorcher**	45°36'N; 7°34'E	5.4	January: -3.0	July: 14.3	1187
**Ferret**	45°50'N; 7°01'E	6.3	January: -2.8	July: 15.8	957
**Grande**	45°22'N; 7°16'E	10.0	January: 0.6	July: 19.6	1074
**Gressoney**	45°51'N; 7°48'E	4.2	January: -3.6	July: 12.7	1202
**Lourousa**	44°12'N; 7°16'E	10.4	January: 2.4	July: 19.1	820
**Sessera**	45°40'N; 8°05'E	9.7	January: 0.1	July: 19.4	1800
**Troncea**	44°57'N; 6°57'E	0.8	January: -8.0	July: 9.5	956
**Valletta**	44°10'N; 7°16'E	10.3	January: 2.4	July: 19.0	828
**Veny**	45°46'N; 6°52'E	5.9	January: -2.5	July: 14.9	906

**Table 2.** Sampling effort details of the fourteen surveys carried out in eleven valleys of the western Italian Alps from 2005 to 2012. T = Trapping; AC = Active manual collection; * = data unavailable .

**Valley**	**Year**	**Sampling method**	**Sampling effort**			**Source**
			**Number of sites**	**Occasions for year**	**Traps per site**	
Argentera	2010	T	4	4	8	Chamberlain et al. 2015
Chalamy	2005-06	T and AC	8	*	3	Palestrini et al. 2008b
Chalamy	2007	T	4	12	6	Macagno and Palestrini 2009
Champorcher	2005-06	AC	5	*	*	Palestrini et al. 2008b
Ferret	2005-06	AC	17	1-6	*	Palestrini et al. 2008a
Ferret	2007	T	7	19	3	Negro et al. 2011b
Gressoney	2010	T	4	4	8	Chamberlain et al. 2015
Grande	2010	T	4	4	8	Chamberlain et al. 2015
Lourousa	2008	T	2	5	9	Negro et al. 2011a
Sessera	2010	T	16	5	5	Tocco et al. 2013a
Troncea_B	2011	T	16	4	4-6	Unpublished data
Troncea	2011-12	T	16	6	4-6	Tocco et al. 2013b
Valletta	2008	T	4	5	9	Negro et al. 2011a
Veny	2005	AC	10	1-4	*	Palestrini et al. 2008a

**Table 3.** List of the species present in eleven valleys of the western Italian Alps. The different surveys have been kept separately. 1 = Valletta valley, 2008; 2 = Lourousa valley, 2008; 3 = Troncea B valley, 2011; 4 = Troncea valley, 2011-2012; 5 = Argentera valley, 2010; 6 = Grande valley, 2010; 7 = Champorcher valley, 2005-2006; 8 = Chalamy valley, 2005-2006; 9 = Chalamy valley, 2007; 10 = Gressoney valley, 2010; 11 = Ferret valley, 2005-2006; 12 = Ferret valley, 2007; 13 = Veny valley, 2005-2006; 14 = Sessera valley, 2010.

	**South-West <--------------------------------------------> North-East**													
**Survey**	**1**	**2**	**3**	**4**	**5**	**6**	**7**	**8**	**9**	**10**	**11**	**12**	**13**	**14**
**Geotrupidae Latreille**														
*Anoplotrupes stercorosus* (Scriba)	X	X	X	X		X	X	X	X	X	X	X	X	X
*Geotrupes spiniger* (Marsham)							X	X	X					X
*Geotrupes stercorarius* (Linnaeus)	X	X	X	X		X	X	X	X	X	X	X	X	X
*Tripocopris pyrenaeus* (Charpentier)										X				
*Tripocopris vernalis* (Linnaeus)	X	X						X						
*Trypocopris alpinus* (Sturm & Hagenbach)	X	X	X	X						X				X
**Scarabaeidae: Aphodiinae Leach**														
*Acrossus depressus* (Kugelann)	X		X	X				X	X	X	X	X	X	X
*Acrossus rufipes* (Linnaeus)	X	X	X	X	X	X	X	X	X	X	X	X	X	X
*Agoliinus satyrus* (Reitter)	X	X	X	X	X					X	X	X	X	X
*Agolius abdominalis* (Bonelli)	X	X									X	X		
*Agrilinus constans* (Duftschmid)	X	X												
*Agrilinus convexus* (Erichson)														X
*Amidorus obscurus* (Mulsant & Rey)	X	X	X	X	X		X			X	X		X	
*Amidorus immaturus* (Mulsant)			X	X						X	X	X	X	
*Ammoecius brevis* (Erichson)												X		X
*Aphodius fimetarius* s.l. (Linnaeus)	X	X	X	X	X		X	X	X	X	X	X	X	X
*Bodilopsis rufa* (Moll)	X	X	X	X	X	X	X	X	X	X	X	X	X	X
*Calamosternus granarius* (Linnaeus)								X	X		X		X	
*Colobopterus erraticus* (Linnaeus)	X		X	X		X	X	X	X	X	X	X	X	X
*Coprimorphus scrutator* (Herbst)			X	X					X					
*Esymus pusillus* (Herbst)			X	X							X	X	X	X
*Euheptaulacus carinatus* (Germar)	X	X	X	X	X						X		X	
*Euheptaulacus villosus* (Gyllenhal)							X	X				X		
*Limarus zenkeri* (Germar)								X	X		X	X		X
*Nimbus contaminatus* (Herbst)														X
*Nimbus johnsoni* (Baraud)														X
*Oromus alpinus* (Scopoli)			X	X	X	X	X	X		X	X	X	X	
*Otophorus haemorrhoidalis* (Linnaeus)	X		X	X			X		X		X	X	X	X
*Oxyomus sylvestris* (Scopoli)														X
*Parammoecius corvinus* (Erichson)	X	X		X	X	X	X	X		X	X	X	X	X
*Parammoecius pyrenaeus* (Jacquelin Du Val)			X							X				
*Planolinoides borealis* (Gyllenhal)														X
*Planolinus fasciatus* (Olivier)	X		X	X	X	X	X	X		X	X	X	X	
*Rhodaphodius foetens* (Fabricius)	X		X	X				X	X		X	X	X	X
*Sigorus porcus* (Fabricius)								X	X					
*Teuchestes fossor* (Linnaeus)			X	X			X	X	X	X	X	X	X	X
*Volinus sticticus* (Panzer)									X					
**Scarabaeidae: Scarabaeinae Latreille**														
*Euoniticellus fulvus* (Goeze)								X	X					X
*Onthophagus baraudi* (Nicolas)	X	X	X	X	X							X		
*Onthophagus coenobita* (Herbst)									X					
*Onthophagus fracticornis* (Preyssler)	X	X	X	X	X	X	X	X	X	X	X	X	X	X
*Onthophagus joannae* (Goljan)								X	X					X
*Onthophagus lemur* (Fabricius)								X						
*Onthophagus opacicollis* (Reitter)														X
*Onthophagus taurus* (Schreber)									X					X
*Onthophagus vacca* (Linnaeus)								X	X					
